# Cytotoxic effect of albumin coated copper nanoparticle on human breast cancer cells of MDA-MB 231

**DOI:** 10.1371/journal.pone.0188639

**Published:** 2017-11-29

**Authors:** Marzieh Azizi, Hedayatollah Ghourchian, Fatemeh Yazdian, Fariba Dashtestani, Hojjat AlizadehZeinabad

**Affiliations:** 1 Institute of Biochemistry and Biophysics (IBB), University of Tehran, Tehran, Iran; 2 Nanobiomedicine Center of Excellence, Nanoscience and Nanotechnology Research Center, University of Tehran, Tehran, Iran; 3 Faculty of New Science and Technology, University of Tehran, Tehran, Iran; University of South Alabama Mitchell Cancer Institute, UNITED STATES

## Abstract

**Purpose:**

The aim of this study was to design a new nanocomposite that would have high cytotoxicity against invasive breast cancer cells and minimum side effects on normal cells.

**Methods:**

An albumin nano-carrier for delivery of CuNPs was developed. The ACuNPs formation was characterized by TEM, DLS and UV-Vis, fluorescence and circular dichroism spectroscopy. The cytotoxic efficacy of the ACuNPs against human breast cancer cells (MDA-MB 231) and normal cells (MCF-10A) was compared using a standard MTT assay. The mechanism of cell death induced by ACuNPs was considered by inverted and fluorescent microscopy, flow cytometry and gel electrophoresis. The effects of compounds on ROS generations in MDA-MB 231 cells were also studied.

**Results:**

It was found that the resulted ACuNPs with a diameter of 62.7 nm and zeta potential of about -10.76 mV, are suitable for extravasation into tumor cells. In ACuNPs, the 90% of the secondary structure and almost all the tertiary structure of albumin remained intact. Comparing to CuNPs, ACuNPs could significantly suppress the viability of cancer cells while they were less toxic on normal cells. Compared with the untreated cells, the MDA-MB 231 cell line showed higher levels of ROS production after treatment with ACuNPs. The increase in ROS production after 24 hours indicated that ACuNPs induce apoptosis.

**Conclusions:**

The ACuNPs characteristics such as intact structure of albumin, high toxicity against cancer cells comparing to normal cells and apoptosis induction as the mechanism of cell death, revealed that this nanocomposite is a good candidate to be used as a chemotherapeutic agent against invasive breast cancer cells.

## 1. Introduction

Among different types of breast cancers, the less and moderately invasive types could be treated by conventional therapeutic method; conversely, there is no treatment for most invasive types yet. Therefore, finding an efficient, biocompatible and cost-effective therapeutic agent against the most invasive breast cancers is a serious challenge from the clinical point of view [1]-[2].

It is worth mentioning that Cu based products have been approved for human usage by US Environmental Protection Agency since February 2008 [3]. This approval could be due to the fact that Cu is an essential trace element with the vital role in abundant metabolic and physiological processes of human beings. Because of its bioactivity, it is increasingly being used in the production of copper-based nanoparticles. Furthermore, Cu nanoparticles (CuNPs) have particularly shown high toxicity against tumor cells such as pulmonary adenocarcinoma (A549) and human leukemia monocytic cell lines (THP-1) [4] [5]. It was shown that the cytotoxic effect of CuNPs in nano-scale is more effective than that in micro-scale [6]. Therefore, it seems that the CuNPs based products in nano-scale have the potential to be used as the chemotherapy drug. On the other hand, it is considered as a general rule that the apoptosis inducing agents are the only cytotoxic molecules that can be used as chemotherapeutic drugs [7].

Apoptosis is a type of cell death with the programmed sequence of events that cause cell mortality without releasing harmful substances toward the adjacent cells. Apoptosis normally occurs during differentiation and development, also it has an important role in response to a variety of environmental stress such as cytotoxic agents and removal of tumor cell [8]. Cytotoxic drug-induced cells damage, particularly nuclear changes, activates apoptosis via either the intrinsic or extrinsic mechanism [8]. One of the observed symptoms in treated cells with anticancer drugs is generation of reactive oxygen species (ROS) [9]. The thus produced ROS has dual roles: induction of cell proliferation in the normal situation and apoptosis induction in the stressed condition [10].

To design an effective chemotherapeutic drug, it is absolutely essential to target cancer cells with minimal toxicity toward the normal cells. Albumin nanoparticles as carriers for targeted delivery of chemotherapeutic drugs, have attracted much attention due to the fact that they increase endocytic uptake of the drugs [11] by rather cancer cells than normal cells. This is firstly due to the enhanced permeation and retention effect (EPR phenomenon) of albumin nanoparticles mediated by the passive uptake of albumin in the tumor cells. Secondly, albumin nanoparticles enhance active absorption of a drug by the tumor cells via albumin receptor. Consequently, a variety of drug delivery systems based on albumin have been attempted including albumin-binding drug derivatives, drug-albumin conjugates, prodrugs and albumin nanoparticles [12]. Another advantage of albumin nanoparticles is the elimination of cremophor and ethanol as organic solvents as well as emulsifiers due to the increased drug solubility [13]. Serum albumin, as the most abundant blood protein has many important functions including maintenance of blood pH, osmotic pressure, and transportation of different types of endogenous and exogenous molecules [14]. Features such as various binding sites for a large number of drugs, high half-life in the blood circulation, great solubility and stability, albumin has attracted considerable attention. Because of more than 70% structural similarity, bovine serum albumin (BSA) has been used as an alternative for human serum albumin (HSA). Furthermore, it is biocompatible, stable, and readily accessible at a much lower price as compared with HSA [15, 16].

In the present report, the novel albumin-based nanocarrier of CuNPs abbreviated as ACuNPs was synthesized. The structural properties of ACuNPs were studied via different spectroscopy methods. Then, their anticancer properties against MDA-MB 231 as invasive breast cancer cells and MCF-10A as normal counterpart were considered. Then, the mechanism of cell death in MDA-MB 231 treated by ACuNPs was followed by studying: 1) cell morphology, 2) nuclear characteristics, 3) DNA ladder formation, 4) annexin V/7-AAD staining monitored by flow cytometry and 5) cell cycle arrest. To investigate the probable triggering factor of apoptosis, the concentration of ROS was determined showing that ACuNPs leads to excel the oxidative stress by a dose-dependent pattern. Overall, our results demonstrate that, compared to CuNPs, the synthesized ACuNPs is a much more potent drug to induce programmed cell death in the cancer cells.

## 2. Materials and method

### 2.1 Materials

BSA (fraction V, minimum 98%) was purchased from Sigma (Steinheim, Germany). Dulbecco's Modified Eagle's Medium (DMEM) and serum albumin of fetal bovine (FBS) were purchased from Gibco (United States); 3-(4,5- dimethyl-thiazol-2-yl)-2,5-diphenyl tetrazolium bromide (MTT), 2',7'-dichlorofluorescein diacetate (DCFHDA), Cu(NO_3_)_2_.5H_2_O, streptomycin, and penicillin was obtained from Sigma (United Kingdom); and dimethyl sulfoxide (DMSO) and trisodium citrate was supplied by Merck (Germany).

### 2.2 Synthesis of CuNPs, ACuNPs, and ANPs

The CuNPs were synthesized by using chemical reduction method [17]. Briefly, 2.4 mg of Cu(NO_3_)_2_.5H_2_O was dissolved in 50 ml double distilled water, heated for 15 min. 5 ml of 1% trisodium citrate was added dropwise. The solution was mixed vigorously and heated until solution color was changed into pale blue. The appearance of bluish color in the reaction vial and lowering the pH from 7 to 5.5 indicate the CuNPs formation. The solution was removed from the heat and stirred until it cooled down to room temperature. The chemical reaction is as follows (Eq 1):

$$2Cu^{+}+C_{6}H_{5}O_{7}{Na}_{3}+2H_{2}O\to 2Cu^{0}+C_{6}H_{5}O_{7}H_{3}+3Na^{+}+H^{+}+O_{2}↑$$ (1)

The ACuNPs were synthesized by using chemically modified nanoprecipitation method [18]. 20 μl absolute ethanol was dissolved in 230 μl dichloromethane. This solution was added to 1 mM CuNPs in 1000 μl of BSA solution (5% w/v) saturated by dichloromethane to produce ACuNPs. It was mixed at low speed for 5 min. Afterward high-pressure homogenization was applied (Bandelin Sonopuls ultrasonic homogenizer, Bandelin electronic, Berlin, Germany). The emulsification was performed at 20 kHz while recycling the emulsion for at least 20 cycles. This resulted in the formation of an opalescent suspension spontaneously at room temperature. The dichloromethane of resultant was rapidly removed at 40°C at reduced pressure (30 mm Hg) for 1 h in a rotary evaporator. The dispersion was further freeze-dried for 24 h. The procedure for synthesizing the blank nanoparticle of albumin (ANPs) was exactly the same as that for ACuNPs synthesis, except that BSA solution was free of CuNPs.

### 2.3 Characterization of ACuNPs

The morphology of ACuNPs was observed by TEM (JEOL JEM 1400Plus equipped with Ruby camera at 120 kV). Then the particles were characterized by UV–Vis spectroscopy using Carry 100 Bio, Varian, Australia equipped with Cary Win UV software. Dynamic light scattering (DLS, Zetaplus; Brookhaven Instruments, Holtsville, NY) was used to measure the average particle size and size distribution of the nanoparticles [19]. The ACuNPs were diluted in PBS and filtered to avoid any dust pollution. To identify surface charge, the zeta potential of the ACuNPs was determined using a Zetaplus zeta potential analyzer (Brookhaven Instruments) at room temperature. In all spectroscopic characterization, the concentration of samples was 40 mM in PBS (0.1 M, pH 7.4). The CD spectroscopy was done with an Aviv spectropolarimeter, model 215 (Lakewood, NJ, USA) with a 1.0 cm and a 0.1 cm path length rectangular quartz cells controlled by a thermoelectric cell holder (Aviv). Fluorescence spectrophotometer (Varian Cary Eclipse) was used to measure the intrinsic fluorescence spectra of BSA at 25°C with the excitation at 295 nm [19, 20].

### 2.4 Cell culture and animal

Highly invasive human breast cancer cell line of MDA-MB-231 (progesterone and estrogen receptor-negative), was purchased from the National Cell Bank of Iran (Pasteur Institute of Iran, Tehran, Iran). MCF-10A, a non-tumourigenic human breast epithelial cell line was obtained from human and animal cell bank at Iranian Biological Resource Center (IBRC). MDA-MB-231 cells were grown as monolayer cultures in DMEM culture medium supplemented with 10% FBS, 100 U/ml penicillin and 100 μg/ml streptomycin. HuMEC ready medium was used for cultivation of MCF-10A. Both cell lines were maintained at 37°C in a humid atmosphere containing 5% CO_2_. Cells were routinely sub-cultured using 0.25% trypsin-EDTA solution (Bio-Idea). The cells that were used for all experiments were in the exponential growth phase [19]. Female BALB/c albino mice (six to eight weeks old with 20±2.0 g body weights) were obtained from Tehran Small Animal Research and Teaching Hospital, Faculty of Veterinary Medicine, University of Tehran, Iran. All mice were kept in pathogen free environment, in polycarbonate cages at 22±2°C temperature, under medical care, 85% relative humidity and a 12-h light–darkness cycle with standard food and water available. They were allowed to adapt to the laboratory conditions for 1 week before treatment. The cages were cleaned at regular interval. All methods and experiments were performed in accordance with the standard ethical guidelines and approved by Animal Care and Ethics Committee of the University of Tehran.

### 2.5 MTT cell viability assay

Measuring the cell viability as criteria for estimation of the cellular growth of treated cells versus controls was carried out using the MTT-based assay according to Mosmann (1983) with slight modifications [21]. A number of 8×10^4^ cells/well were cultured in 96-well plates and kept at 37°C, 5% CO_2_ for 24 h. Then, different concentrations of CuNPs and ACuNPs were added to each well in triplicate and kept at 37°C in a CO_2_ incubator for 24 h again. After that, 20 μl of MTT solution (5 mg/ml in PBS) was added to each well and incubated for 3 h. The supernatant of each well was thrown away and 100 μl of DMSO was added to the attached cells. The absorbance of each well at 570 nm was measured by an ELISA plate reader (Bio-Tek Instruments, USA). The percentage of cell viability was calculated based on Eq 2 [21]:

$$Cell\;Viability\;(\%)=100\times \frac{OD_{sample}}{OD_{control}}$$ (2)

**Fig 1:**
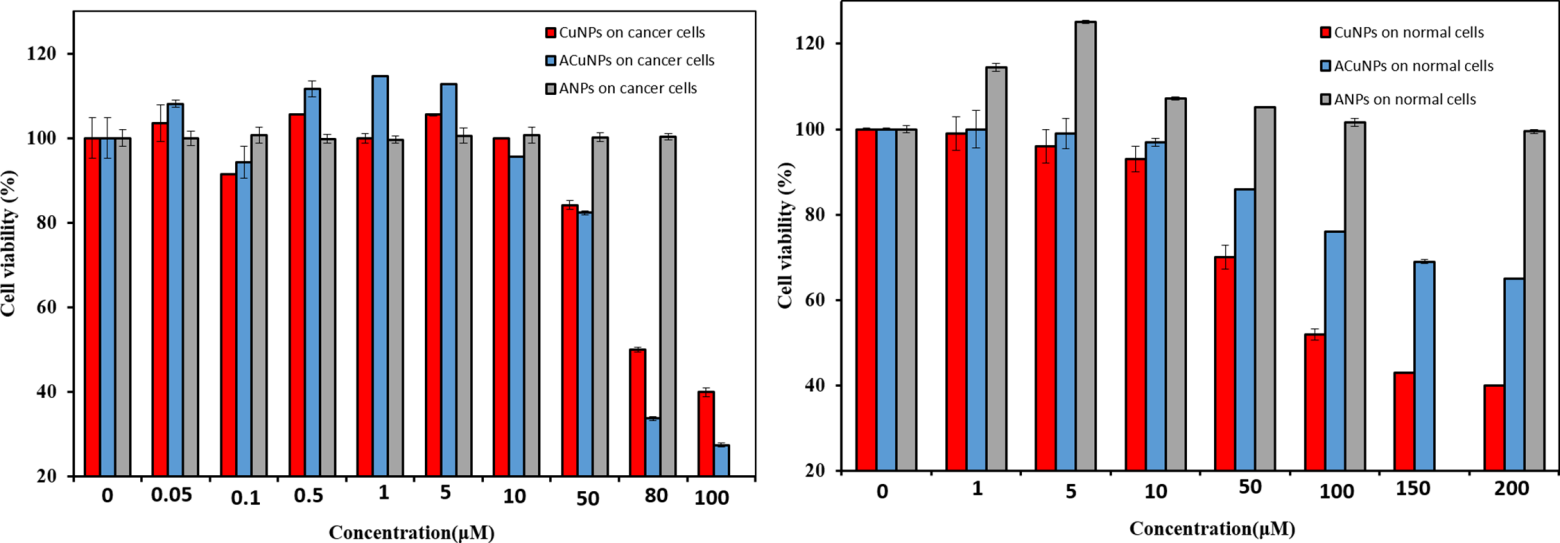
Percent of cell viability after treating by ANPs (grey), CuNPs (red) and ACuNPs (blue) related to MDA-MB 231 cells (A) and MCF-10A cells (B).

The concentrations of CuNPs and ACuNPs at which the cell viability is reduced by half (IC_50_) was calculated based on the resulted curve in Fig 1 [21].

### 2.6 Cytomorphological changes in MDA-MB 231

MDA-MB 231 cells (5×10^5^ cells/well) were seeded in 6-well plates and kept at 37°C, 5% CO_2_ for 24 h. Then, they were treated with IC_50_ of CuNPs and ACuNPs and again incubated for 24 h at 37 ^o^C, 5% CO_2_. After the incubation of cells, the visible morphological changes in the cells were pictured under an inverted phase contrast microscopy (Zeiss, Germany).

### 2.7 Nuclear characteristics in MDA-MB 231

Both of acridine orange (AO) and ethidium bromide (EB) are DNA specific dyes. AO could stain both live and dead cells. EB will penetrate only in necrotic cells with porous membrane and make them red. Live cells will be seen homogeneously green because of AO penetration. Cells in the early apoptotic stage are bright green due to the cleavage of chromatin DNA into internucleosomal fragments and also chromatin condensation. Orange stain with internucleosomal fragments or condensed DNA is related to late apoptotic cells because of EB absorption. Orange stain without apoptotic nuclei shows necrotic cells. MDA-MB 231 cells (5×10^5^ cells/well) were seeded in 6-well plates and kept at 37°C, 5% CO_2_ for 24 h. Then, they were treated with IC_50_ of CuNPs and ACuNPs and again incubated for 24 h at 37°C, 5% CO_2_. Then the cells were dissociated using trypsin and were washed with PBS. After that, 25 μl of the cell were incubated in 1 μl of AO/EB mixture. Finally, 10 μl of the stained cells were placed onto a microscopic slide and covered with a glass coverslip. It was visualized under a fluorescence microscope (Zeiss, Germany).

### 2.8 DNA fragmentation assay

MDA-MB 231 cells (5×10^6^ cells/well) were seeded in 60×15 mm SPL cell culture dishes and kept at 37°C, 5% CO_2_ for 24 h. Then, they were treated with IC_50_ of CuNPs and ACuNPs and again incubated for 24 h at 37°C, 5% CO_2_. Subsequently, the cells were dissociated using trypsin. DNA extraction was done using salting out method. Briefly, the detached cells were washed by cool PBS and lysed with 3 ml of lysis buffer (400 mM NaCl, 10 mg/ml proteinase K, 20 mM Tris- HCl, pH 8.0, 5 mM EDTA and 1% SDS) for 1 h at 55°C. The chromatin DNA was purified by adding 2 ml of 6 M NaCl. After centrifugation, the supernatant was transferred into a new sterile 1.5 ml microtube for the next precipitation step using cold isopropyl alcohol. After washing with ethanol 70%, the pellet was diluted in 100 μl doubled distilled H_2_O and stored at -20°C. The extracted DNA was run on a 2% agarose gel for 20 min at 100 V. At the end of electrophoresis, the gel was stained with ethidium bromide. The bands were visualized with a Kodak Digital Science DC-40™ digital camera in an ultraviolet transilluminator [22].

### 2.9 Flow cytometry based cell apoptosis assay

Apoptosis analysis using flow cytometry was used to quantitatively determine apoptosis and necrotic cell population. An early marker of apoptosis is protrusions of phosphatidylserine (PS) to the outer leaflet of the plasma membrane. Phycoerythrin (PE) annexin-V is a fluorescent-labeled dye which can bind very specifically to PS on the surface of cells, leading to detection of early apoptotic cells. The loss of membrane integrity which is preceded by PE Annexin-V staining shows the latest stages of cell death consequential from either apoptotic or necrotic processes. 7-Amino-Actinomycin (7-AAD) is a vital dye to allow detection of early apoptotic cells (7-AAD negative). Viable cells with intact membranes reject 7-AAD, while the membranes of damaged and dead cells absorb it. Therefore, annexin-V and 7-AAD negative cells are viable, PE annexin-V positive and 7-AAD negative cells are early apoptotic cells and both PE annexin-V and 7-AAD positive cells are late apoptotic, and PE annexin-V negative and 7-AAD positive cells are necrotic cells. The degree of apoptosis in cells exposed to CuNPs and ACuNPs was quantified by PE annexin-V and 7-AAD staining using flow cytometry. After incubation, 300 μl binding buffer was added to each sample, and cells were kept on ice. Data analysis was performed using Flowjo software, version 7.6.1 [19].

### 2.10 Flow cytometry based cell cycle assay

Propidium iodide (PI) was used for DNA staining to determine cell cycle phase. Approximately 6×10^6^ cells/well of MDA-MB-231 cells were seeded in 60×15 mm SPL cell culture dishes and kept at 37°C, 5% CO_2_ for 24 h. Then, the cells were treated with IC_50_ of CuNPs and ACuNPs and again incubated for 24 h at 37°C, 5% CO_2_. The cells were detached, centrifuged, washed with PBS, fixed in 70% cold ethanol, and stored at 4°C for at least 12 h. The cells were washed with PBS and treated by PI in RNase (40 μg ml^−1^ of PI in 100 μg ml^−1^ of RNase A) and incubated at 37°C for 30 min. Flow cytometry analysis was performed using FACS Calibur at an excitation wavelength of 488 nm and an emission wavelength of 610 nm. The data collected for 10000 cells were analyzed using Flowjo software.

### 2.11 Cellular ROS assay

The level of intracellular ROS was assayed by a fluorescent dye of DCFHDA. After entering in to the cell, DCFHDA would be oxidized by ROS into a fluorogenic substrate named as 2’,7’-dichlorofluorescein (DCF). There is a direct relationship between fluorescence intensity of DCF and the level of intracellular ROS [22]. DCF is detected via fluorescence spectroscopy with excitation and emission wavelength of 495 and 525 nm, respectively [23]. In order to assay the intracellular ROS, approximately 3.5×10^5^ MDA-MB-231 cells/well were seeded in 24-well plates and kept at 37°C, 5% CO_2_, for 24 h. Then, they were treated with CuNPs and ACuNPs for 24 h at the same conditions. As for ROS positive control, a freshly prepared hydrogen peroxide (0.1 mM in PBS) solution was used. 10 μM DCFHDA was added to each well of treated cells and incubated at 37°C, 5% CO_2_, for 1 h followed by twice washing with PBS. 500 μl PBS was added to each well and homogenized carefully. Finally, the fluorescent intensity was measured [22].

### 2.12 In vivo toxicity tolerance and anticancer efficacy

For in vivo study, 25 mice were used. They were divided into 5 groups, with 5 mice in each group. 4T1, a highly malignant of mammary carcinoma cell line of murine, were used to induce cancer in mice (subcutaneously between the two ends of the mammary gland). The first group (normal control group) did not receive any injections; neither 4T1 cells nor ACuNPs treatment. Group 2 was treated only by ACuNPs. Groups 3, 4 and 5 were injected by 1×10^6^ cells/100 μl 4T1 cell line. The third group was used as turmeric control group. Group 4 was treated by 1 mM ACuNPs (100 μl, intraperitoneal injection) when primary tumors reached a mean diameter of 3–4 mm. Group 5 was exposed to a 4T1 cell line and ACuNPs together to study prevention role of from tumor incidence. Groups 4 and 5 were the main treatment groups. It should be noted that molarity of ACuNPs was based on CuNPs concentration, not BSA.

## 3. Results

### 3.1 Characterization of the synthesized CuNPs

**Fig 2:**
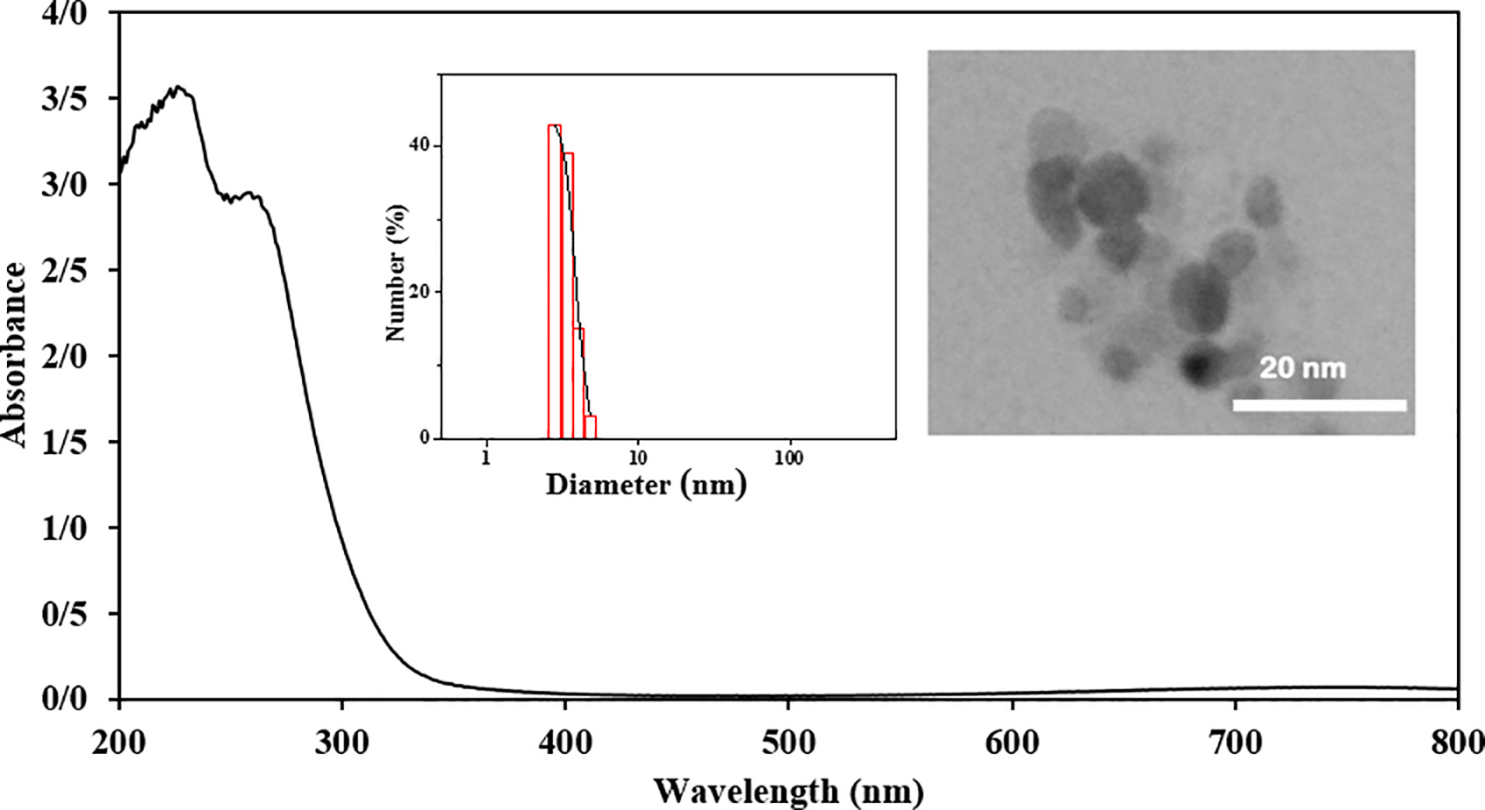
UV-Vis spectrum of 1 mM CuNPs suspension in water. The inset shows DLS plot with PDI of 0.66 (left) and TEM image of CuNPs (right).

To verify the formation of CuNPs, UV–Vis spectroscopy was used. In the spectrum of the synthesized CuNPs, two strong and wide peaks were detected at around 230 and 260 nm (Fig 2). The absorption spectrum of colloidal CuNPs at around 230 and 260 nm is due to the excitation of surface plasmon vibrations (200–300 nm) [24, 25]. The particle size histogram obtained by DLS (Fig 2) indicates that CuNPs vary in size from 2.8 to 4.8 nm (mean diameter of 3.3 nm and the polydispersity index of 0.66). TEM image of synthesized CuNPs shows that they have spherical shapes with diameters around 5 nm (Fig 2).

### 3.2 Characterization of the synthesized ACuNPs

**Fig 3:**
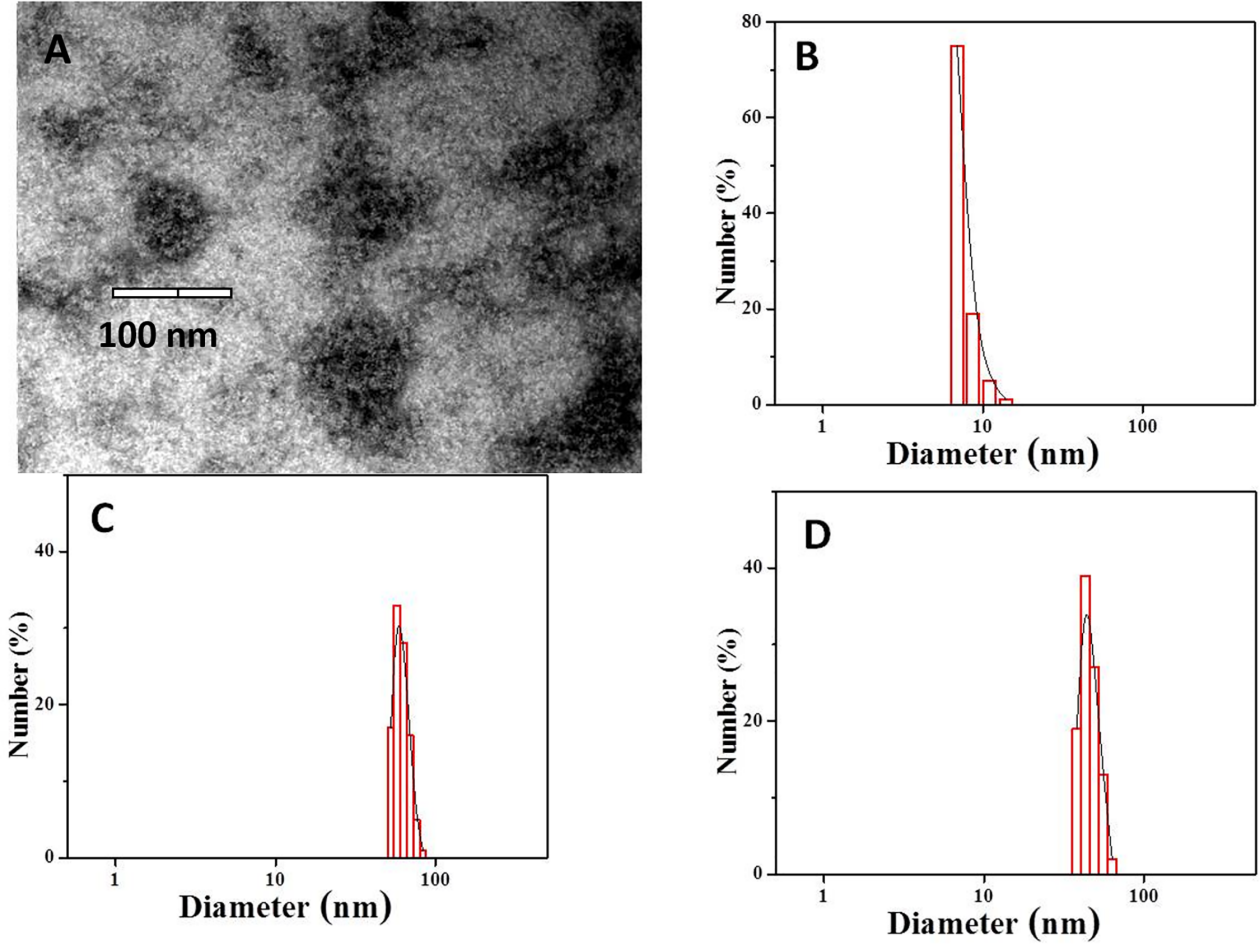
TEM images of negatively stained ACuNPs (A), DLS data of native BSA, PDI: 0.38 (B), ACuNPs, PDI: 0.36 (C) and ANPs, PDI: 0.30 (D).

TEM images of ACuNPs (Fig 3A) revealed 100 nm spherical particles with rather smooth borders containing plenty of black spots inside. It seems that the black spots are CuNPs surrounded by BSA molecules. Since the metallic CuNPs are so condensed, they seem black, but the protein molecules around them seem rather transparent. DLS graph (Fig 3C) indicates that the diameter of ACuNPs varies from 52.5 to 83.5 nm (mean diameter: 62.7 nm) with good homogeneity (PDI 0.36). DLS results verify the information obtained by TEM.

On the other hand, considering the fact that an albumin molecule has an effective diameter of 7.2 nm (Fig 3B), and the diameter of CuNPs is around 3.3 nm, one may conclude that each ACuNPs contain several CuNPs and BSA molecules. ANPs were 46.6 nm in diameter (Fig 3D). Therefore, at least 7 molecules of BSA are engaged in each ANPs. According to the same condition for synthesizing ANPs and ACuNPs, increasing the diameter from 46 to 62 nm is a result of embedding CuNPs inside BSA nanoparticle.

The yield percentage of the synthesized ACuNPs (y) was calculated using Eq 3 as 96%.

$$y=100\times \left(\frac{Weight\;of\;ACuNPs}{Total\;weight\;of\;BSA+CuNPs}\right)$$ (3)

The reason for such a high yield is that no washing was performed in the synthesis process, and therefore; all raw materials are converted into products. This high yield suggests that the synthesis of the nanoparticles is economically efficient, and we estimate that their industrial production efficiency might even be higher. The 4% loss in yield is related to the loss of material during displacement between containers, which could be minimized in industrial-scale.

#### 3.2.1 Zeta potential

The zeta potential could be considered as a significant indicator for showing the stability of the synthesized ACuNPs. The magnitude of zeta potential indicates the degree of electrostatic repulsion between adjacent nanoparticles. D. Hanaor and R. W. áO'Brien reported that particles with charges from ±10 to ±30 are generally found to be stable [26, 27].

The zeta potential for CuNPs was measured to be -16.25 ± 0.53 mV, which is probably due to citrate anions. This charge seems to be large enough to protect the particles from aggregation. Moreover, in the case of ACuNPs, since the small CuNPs are embedded inside the large BSA clusters, the dominant potential of ACuNPs would still be negative. The isoelectric point value of BSA is 4.7 therefore it is negatively charged at pH 7.4. So, the zeta potential for ACuNPs was measured to be -10.76 ± 0.51 mV.

#### 3.2.2 Absorption spectroscopy

**Fig 4:**
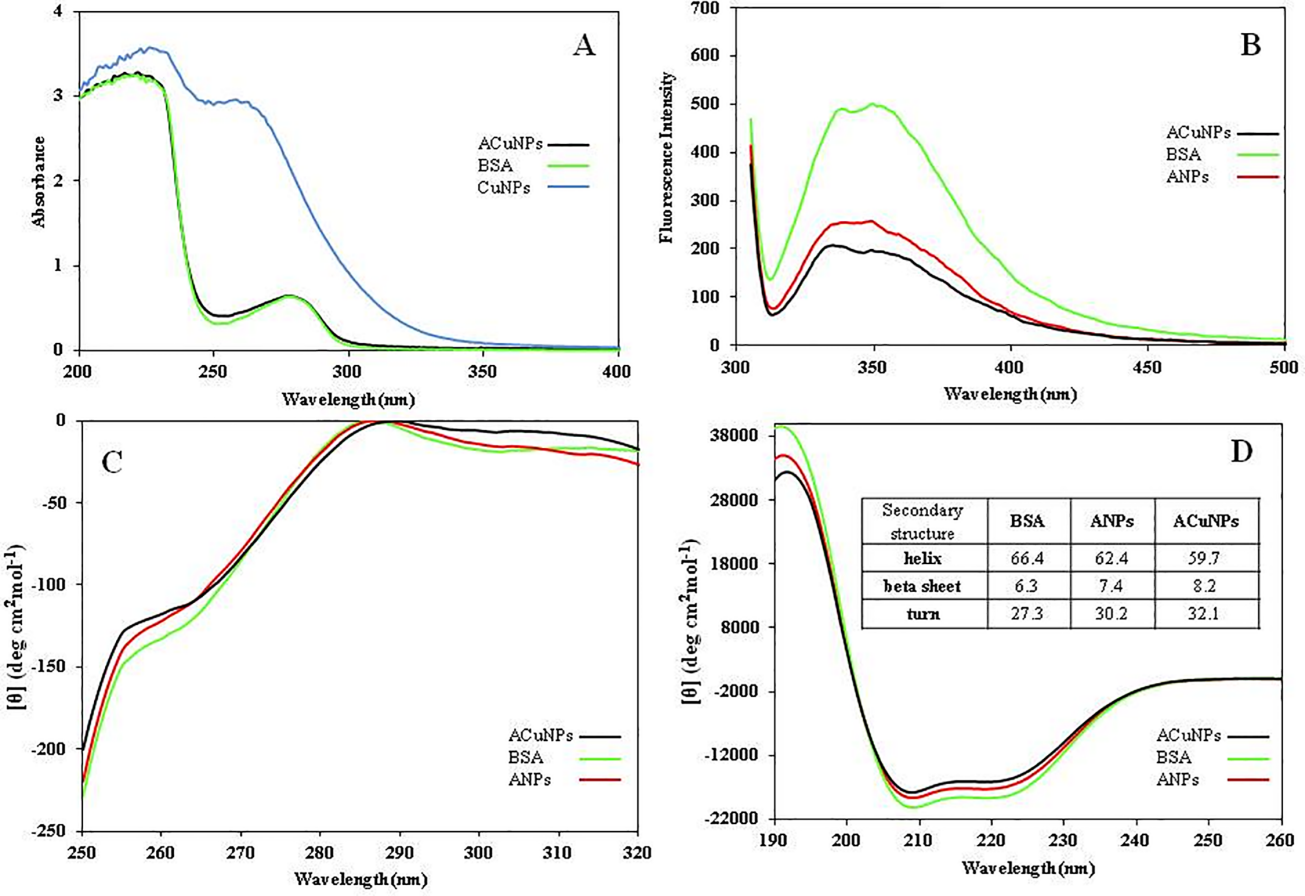
A) UV-Vis, B) Emission fluorescence, C) Far-UV CD and D) Near-UV CD spectra of CuNPs, ACuNPs, ANPs and BSA. The inserted table shows the secondary structural changes.

Embedding of CuNPs in BSA cluster was further confirmed by UV-vis absorption spectra as shown in Fig 4A (solid line). The primary analysis of synthesized nanomaterials by UV–vis spectroscopy has proven to be a very valuable technique for characterization of nanomaterials [28, 29]. The suspension of CuNPs in PBS (1 mM) illustrates two peaks at 230 and 260 nm (Fig 4, triangle-line). These peaks are originated from the surface plasmon resonance of colloidal copper. Fig 4 (dashed line) shows that the strong absorption of BSA near 280 nm is due to the presence of tryptophan [30]. As seen, the UV-visible absorption spectra of BSA and ACuNPs are very close to each other. This indicates that no significant change was happened in the BSA of ACuNPs. On the other hand, the loss of plasmon peak at 260 nm supports the idea that CuNPs are hidden deep in BSA.

#### 3.2.3 Emission spectroscopy

The emission spectrum is informative for molecular environment in the vicinity of tryptophan residues [31]. Fig 4B illustrates the fluorescent emission spectra of ANPs, ACuNPs, and BSA upon excitation at 295 nm. As seen, the emission spectra of ACuNPs and ANPs are close to each other, but BSA shows much higher intensity. The decrease in the emission is related to the deep diving of tryptophan residues. More decreasing of the intensity in ACuNPs relative to ANPs could be due to the exposure of tryptophan residues to the hydrophilic environment produced by metallic nanoparticles (CuNPs). The data obtained from fluorescence spectroscopy is in agreement with molar ellipticity increment in near-UV CD spectroscopy.

#### 3.2.4 Circular dichroism spectroscopy

Evaluation of the extent of the conformational changes of BSA during the process of nanoparticle synthesis was done by CD spectroscopy. As shown in Fig 4D, the α-helix structure in ACuNPs and ANPs are almost similar to those in the native state. This was verified by spectral deconvolution using CDNN program version 2. The results are consistent with 1–2% rise in β-strand and 4–6% reduction in α-helix when the protein is incorporated into nanoparticle assemblies (Fig 4D, Insert table). Since there is such a small change, we may conclude that BSA structure remained almost intact during the process for ACuNPs synthesis.

Furthermore, tertiary structure changes in Fig 4C represents the typical CD spectrum of native BSA which is in agreement with the spectrum reported in the literature [32]. As seen, this spectrum is almost conserved in ACuNPs.

In addition, a shoulder is observed at 255–265 nm of CD spectrum which is the fingerprint of BSA tertiary structure. This fingerprint is visible in all cases of native BSA, ACuNPs, and ANPs suggesting no drastic change happened around tyrosine and tryptophan residues. However, the small increase in the molar ellipticity of ANPs and ACuNPs could be attributed to the slight unfolding which occurs in BSA tertiary structure during the high-pressure homogenization process.

### 3.3 Cytotoxicity assay

As observed in Fig 1A and 1B, ANPs did not exert toxicity on cancer and normal cells, so they could have the potential to act as non-toxic drug carriers for cells. The effect of CuNPs on cancer cells showed that they induced cytotoxic effect at higher concentration (IC_50_ 80 μM). This could be due to the fact that copper is an essential micronutrient involved in all major metabolic pathways, but at higher concentrations, copper may interfere with a variety of cell functions including catalytic, structural and regulatory activities, resulting in inhibition of proliferation (Fig 1A). The effect of CuNPs on normal cells (Fig 1B) showed only dose-dependent cytotoxic effect with IC_50_ of 109 μM. The pattern of ACuNPs effect on cancer cells (Fig 1A) was similar to that of the CuNPs with IC_50_ of 70 μM. The cytotoxic effect of both CuNPs and ACuNPs against MDA-MB 231 breast cancer cells was dose-dependent. The cytotoxicity of copper nanoparticles on cancer cell is accelerated by placing CuNPs in albumin nanoparticles carrier, possibly due to the increased uptake of the CuNPs. Assaying side effect of ACuNPs was done by an MTT cytotoxicity test on MCF-10A, a non-tumourigenic human breast epithelial cell type as a control cell line (Fig 1B). The results showed that ACuNPs inhibited the proliferation of human normal MCF-10A breast cells at higher concentration than the 400 μM concentration at which they inhibited the cancer cells. In fact, the presence of BSA as the carrier of CuNPs intensified the difference between cytotoxicity effect in cancer and normal cells. Cytotoxicity of ACuNPs against breast cancer cells was 5.7 times more severe than their cytotoxicity against normal cells, while this difference for CuNPs was 1.4 times. It seems that ACuNPs facilitate better specific targeting of CuNPs for tumor cells, resulting in less toxic effects on non-cancerous cells. The proposed dose of ACuNPs (70 μM) is not toxic to normal cells. Consequently, it seems that bonding to BSA has resulted in the specific uptake of the ACuNPs by cancer cells. This conclusion is in agreement compatible with what was reported by Furthermore, S. Honary and F. Zahir (33). They reported that negatively charged nanoparticles have lower cytotoxicity compared to positively charged particles, a statement that. This was also approved by FDA [33]. Therefore, ACuNPs have suitable charge that makes them excellent candidates to be used in chemotherapy.

#### 3.3.1 Cytomorphological changes on MDA-MB 231 cells

**Fig 5:**
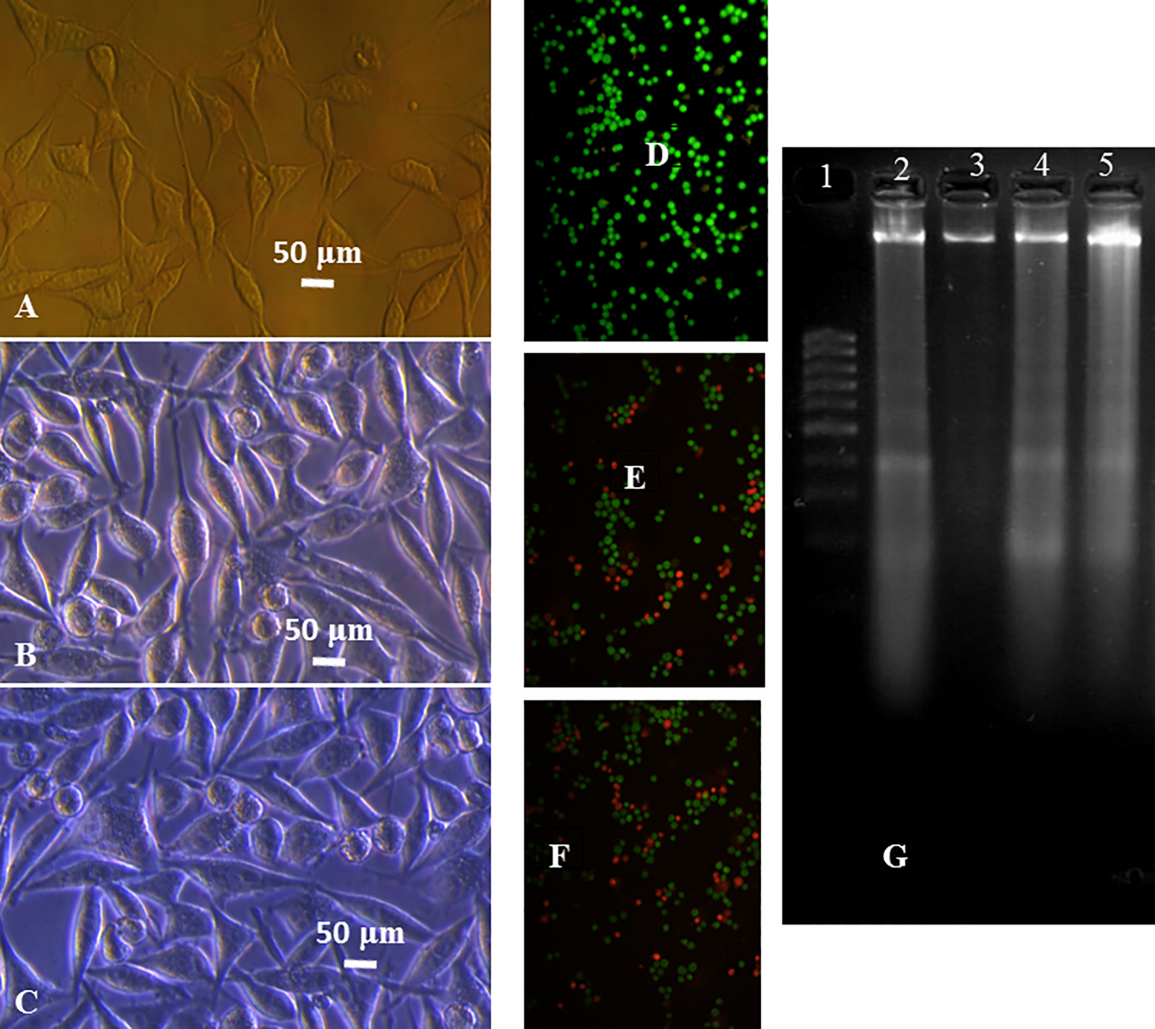
Left: Inverted microscopic images of MDA-MB-231 cells. A: control, B and C: CuNPs and ACuNPs treated cells at IC_50_ concentrations for 24 h, respectively. Middle: Fluorescent microscopic images of AO/EB stained MDA-MB-231 cells. D: control, E and F: CuNPs and ACuNPs treated cells at IC_50_ concentrations for 24 h, respectively. Right: Agarose gel electrophoresis image of DNA extracted from MDA-MB-231 cell line treated with CuNPs and ACuNPs at IC_50_ concentrations (80 and 70 μM, respectively) for 24 h. Lane 1: 1 kb ladder as the marker, lane 3: the untreated cells as the control, lanes 1 and 4: the cells treated with CuNPs and ACuNPs, respectively.

ACuNPs treated cancer cells showed several morphological transformations compared to untreated cells. Such transformations were also seen in CuNPs treated cells. These included inhibition of cell growth, loss of membrane integrity, cytoplasm condensation, creating apoptosis body, and curved forms (Fig 5A–5C).These changes induced apoptotic cell death in cancer cells, whereas the non-treated cells were alive.

#### 3.3.2 Nuclear characteristic in MDA-MB 231

For further improvement of apoptotic cell death in CuNPs and ACuNPs treated cells, AO/EB double staining method was used (Fig 5D–5F). Apoptosis is distinguishable based on nuclear color changes. Orange particles were related to the apoptotic cells because of permeability for both AO and EB, while green particles were related to the viable cells due to impermeability toward EB. The treated cells, illustrated a large number of apoptotic cells compared to control cells.

#### 3.3.3 DNA fragmentation

To distinguish between the apoptotic and necrotic cells, the biochemical technique of DNA fragmentation analysis was conducted as a cellular marker for apoptosis. This assay involves extraction of DNA from a lysed cell homogenate, followed by agarose gel electrophoresis. The DNA of MDA-MB-231 cells treated with CuNPs and ACuNPs were extracted and loaded on the 2% agarose gel. The results (Fig 5G) show that the pattern of DNA ladder is somehow related to the extracted chromatin from CuNPs and ACuNPs treated cancer cells.

#### 3.3.4 Quantification of apoptosis

**Fig 6:**
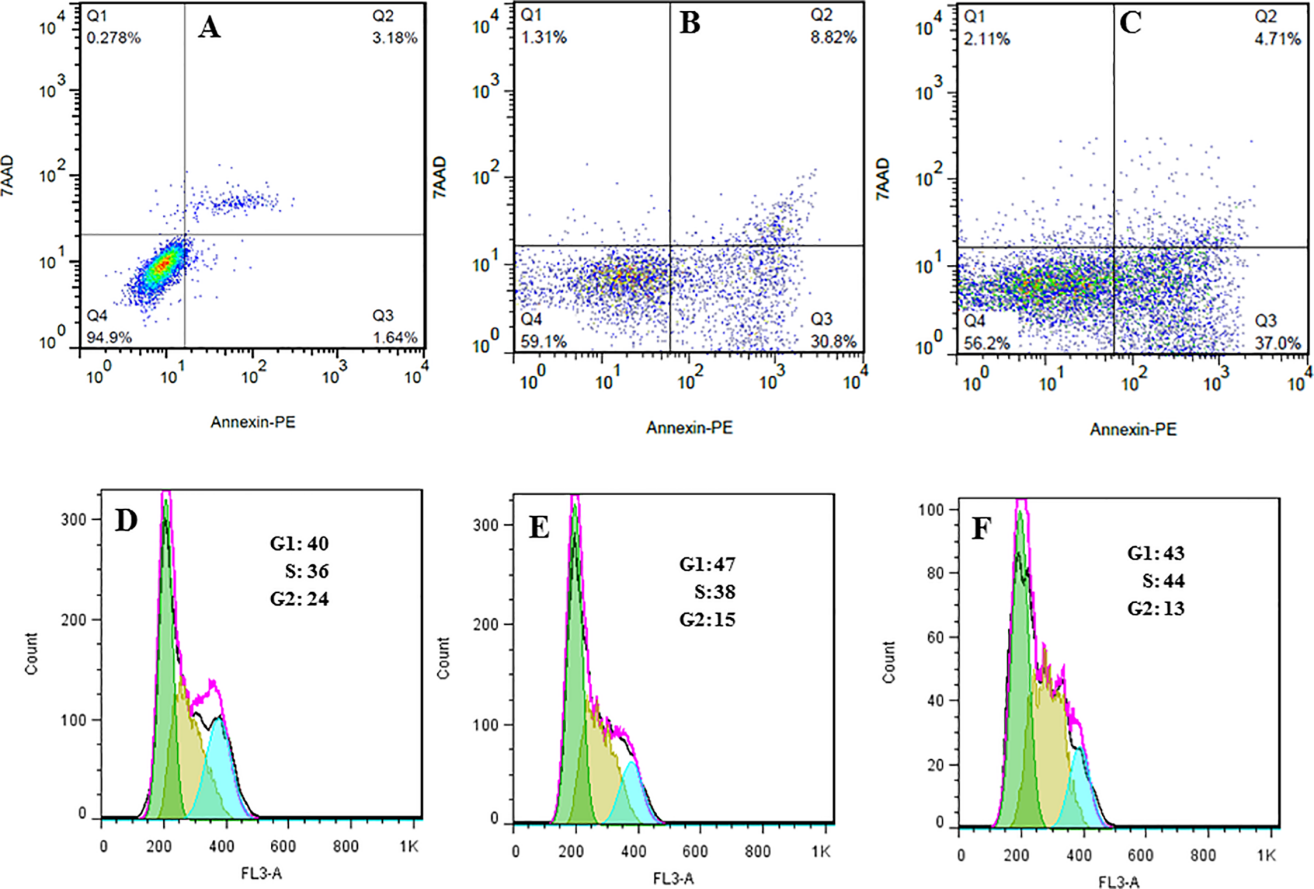
(A-C) Two-dimensional contour density plots of MDA-MB-231 cells obtained by flow cytometry based assays. A: Control, B and C: Treated cells with CuNPs and ACuNPs, respectively. Cell necrosis and apoptosis were measured using 7-AAD and Annexin-V dyes. D-F: Flow cytometry-based assay of the cell cycle. D: Control, E and F: Treated cells with CuNPs and ACuNPs, respectively.

Quantification of apoptosis data were performed using flow cytometry. Fig 6A–6C shows the ACuNPs and CuNPs induced apoptosis in MDA-MB 231cells after 24 h treatment at IC_50_ concentrations.

### 3.4. Effect of ACuNPs on cell cycle

The effect of ACuNPs on cell cycle progression in MDA-MB 231 cells was analyzed after exposure to ACuNPs at the concentration of IC_50_ for 24 h. Cells with damaged DNA will accumulate in gap1 (G1), DNA synthesis (S), or in gap2/mitosis (G2/M) phase [34]. Cells with irreversible damage will undergo apoptosis (34). As shown in Fig 6D and 6E, 24 h after ACuNPs treatment at IC_50_ concentration, 43% of the MDA-MB 231 cells were in G1 phase, 44% were in S phase, and 13% were in G2 phase compared to the untreated control cells (40% of the cells in G1 phase, 36% in S phase, and 24% in G2 phase). DNA loss in apoptotic cells, due to cell permeation and fixation in ethanol before staining, may result in releasing of oligonucleosomes and mononucleosomes; therefore, the population of necrotic or apoptotic cells is not visible in cell cycle data of Fig 6D and 6E. The results demonstrated that the pattern of the changes in cell cycle is the same in ACuNPs and CuNPs treated cells. Both of CuNPs and ACuNPs at IC_50_ concentrations resulted in the increase of accumulation in G1 and S phases and decrease of G2 population. This phenomenon is probably due to the fact that the action mechanisms of CuNPs and ACuNPs inside the cells were similar. This is the same pattern of cell cycle changes as that achieved by M. Ahamed, et, al. indicating that CuNPs-induced cell cycle arrest in human breast cancer of MCF-7 cells results in the appearance of significant amount of apoptotic cells and significant decline in G2/M phase [35].

### 3.5. Intracellular ROS increasing during ACuNPs treatment

It has been reported that cytotoxicity of nano-leveled materials is related to intracellular ROS increment [36]. ROS is involved in several biological functions of the cell in both normal or stress conditions. Under normal conditions, ROS triggers growth and development; however, under stressed conditions, ROS may lead to pathological effects, even cell death. Excessive amounts of intracellular ROS lead to the apoptosis [37]. Additionally, it was shown that the mechanism of apoptosis induction under treatment with heavy metal nanoparticle may correlate with increased levels of ROS. This was confirmed where ACuNPs led to increase of the intracellular levels of ROS.

**Fig 7:**
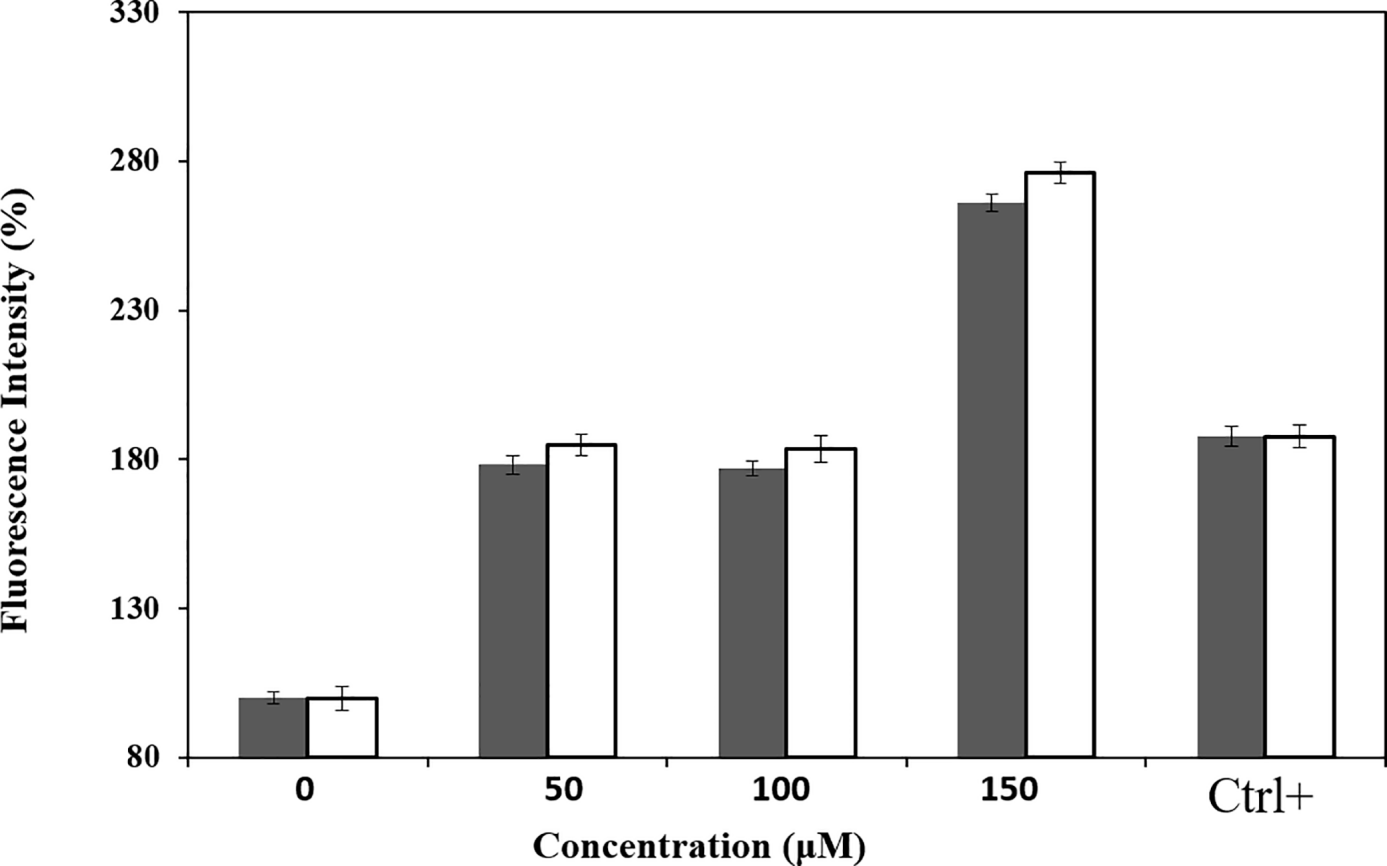
ROS increment in CuNPs ■ and ACuNPs □ treated MDA-MB-231 cells. Relative fluorescence emission of DCF was measured via fluorescence spectroscopy with excitation and emission wavelength of 495 nm and 525 nm, respectively. The first two columns indicate the untreated cells as the negative control and the last two columns are related to the H_2_O_2_ treated cells as the positive control (Ctrl+).

The potential of ACuNPs to increase intracellular ROS level was evaluated using DCFHDA assay. Treated cells with 1mM of H_2_O_2_ were considered as positive controls to validate the test. It would actually confirm that the DCFH-DA, which we used in the test reacts with hydrogen peroxide (H_2_O_2_, which is a well characterized ROS) and emits as expected. 24 hours after the ACuNPs treatment, the emission of the green fluorescent DCF dye was read at 525 nm. As depicted in Fig 7, ACuNPs, like CuNPs led to increase of the oxidative stress in a dose-dependent pattern. It could be concluded that the mechanism of ACuNPs affection on treated cells is through increased ROS. A previous publication has also comprised that copper nanoparticles could increase ROS levels in human breast cancer MCF-7 cells (35).

### 3.6. Effect of ACuNPs on the body weight of mice and tumor size

**Fig 8:**
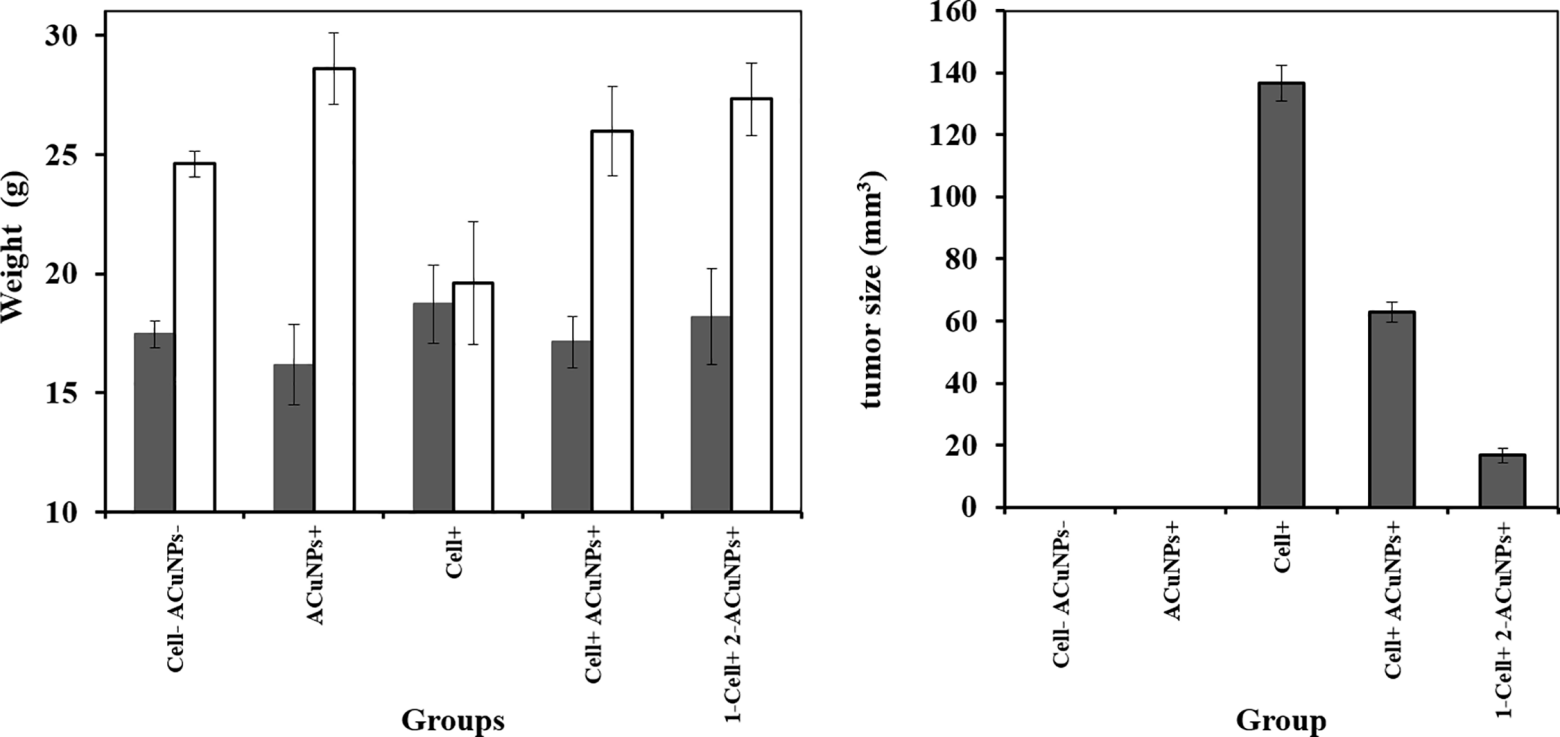
Effect of ACuNPs on the: A) mice’s weight as weight of the animal before the start of the experiment ■ and at end of the treatment □ and (B) tumor size in chemically induced murine tumor by 4T1 cells in BALB/c, the results are expressed as the mean ± error bar. Group 1 was untreated mice as control (without 4T1 cells and ACuNPs injection), Group 2 was treated only by ACuNPs, Group 3 was treated only by 4T1 cells, Group 4 was treated by 4T1 cells and ACuNPs simultaneously and Group 5 were injected by 4T1 cells first, and after 7 days, when primary tumors reached a mean diameter of 3–4 mm, they were treated by ACuNPs.

The mice that were only treated with 4T1 cells could not gain enough weight in 14 days. All groups that were treated with ACuNPs (included: groups 2, 4 and 5) had higher weight than non-receiving ones. Also, group 2, which was injected only with ACuNPs had the highest weight than other groups. These results indicated that ACuNPs have a positive effect on growth of BALB/c mice (Fig 8).

A significant difference in the tumor size is depicted in Fig 8B. As predicted, groups 1 and 2 did not show any signs of tumor. The maximum tumor size was found in group 3, which was injected by only 4T1 without any ACuNPs treatment. Comparison between groups 4 and 5 revealed that ACuNPs could prevent tumorigenesis, but the ACuNPs would be less effective for preventing the tumorigenesis compared to effectiveness as inducers of apoptosis. Fig 8B shows that ACuNPs treatment removes tumor gland in both groups 3 and 4. The strongest reduction of tumor size occurred in group 5 that is associated with ACuNPs-treated tumor-bearing mice.

## 4. Discussion

Albumin nanocarriers have attracted much attention in the field of targeted delivery of therapeutic agents. This is primarily due to their high drug loading capacity, biodegradability, biocompatibility, and the possibility of carrying different types of hydrophobic and hydrophilic drugs.

Furthermore, anti-breast cancer effects of CuNPs have been documented [35]. The proposed mechanism for this effect is the interaction of the CuNPs with the intracellular macromolecules like proteins and DNA. Cellular uptake of the CuNPs leads to increased ROS, resulting in induction of apoptosis [38]. Therefore, ACuNPs were designed and developed for targeted delivery of CuNPs to one of the most invasive breast cancer cell lines, namely MDA-MB-231.

**Fig 9:**
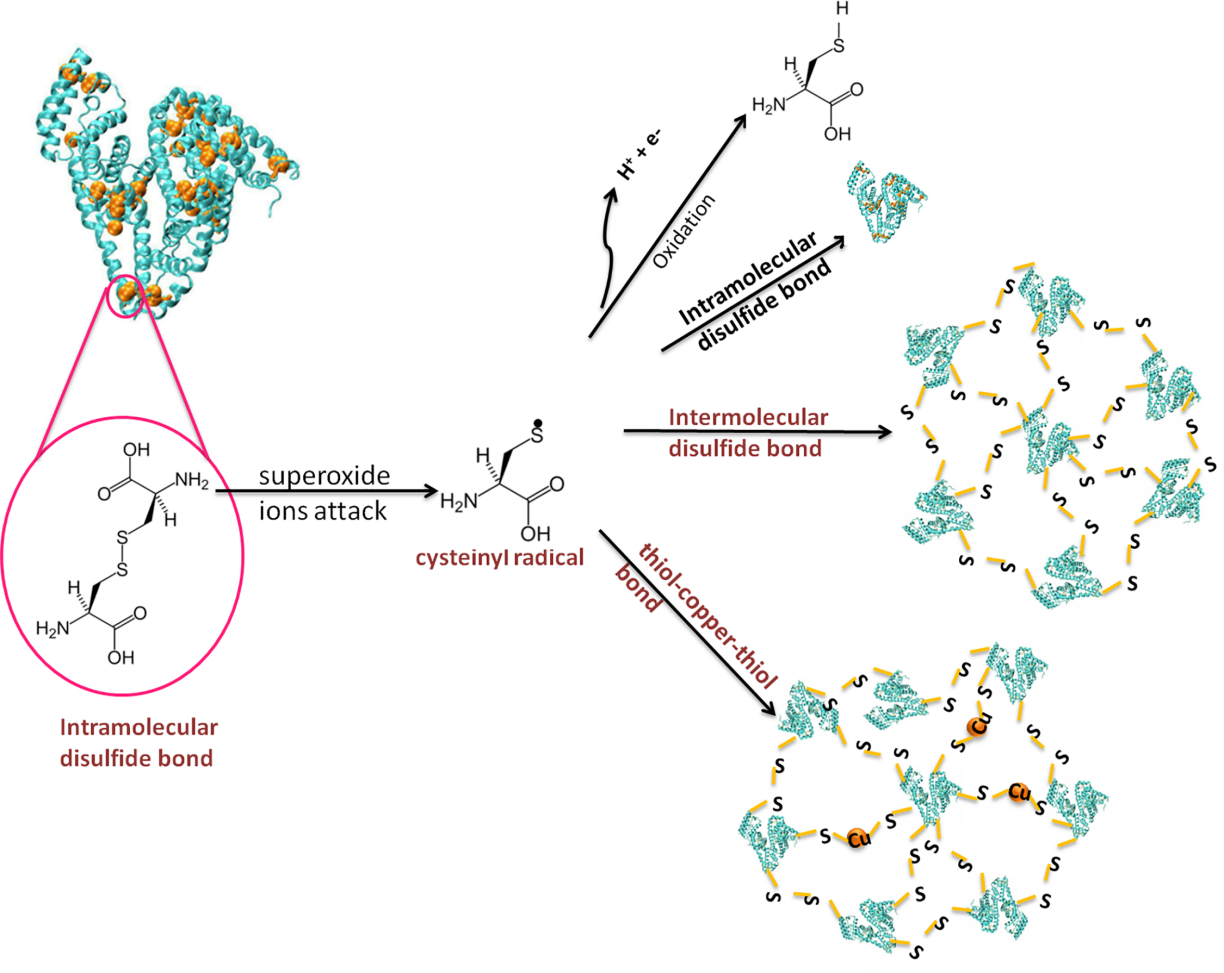
Proposed mechanism of ANPs and ACuNPs formations.

At first, the blank nanoparticles of albumin were synthesized and named as ANPs. To understand the mechanism of ANP formation, we focused on the fact that ultrasonication process produces cavitation in the liquid that causes tremendous local heating and pressure which can lead to the formation of superoxide ions. The thus produced superoxide ions attack BSA and disrupt the existing disulfide bonds between its cysteine residues and produce cysteinyl radicals. In this step, some of the cysteinyl radicals are inhibited by transforming to SH groups in cysteine residues of BSA molecules; and some others join together to reform the intramolecular disulfide bonds at the cysteine residues of each BSA. However, because of their instability, the cysteinyl radicals tend to attack each other and accumulate in the form of ANPs. The idea of ANPs formation comes from the proposal inspired from the patent registered by Desai N.P., et al. [39]. They proposed that the ANPs formation begins by replacing the disulfide bonds within each BSA molecule with such bonds between them. By the accumulation of at least 7 BSA molecules together via formation of new inter-molecular disulfide bonds between cysteinyl radicals in the adjacent BSA molecules, they join to form ANPs (Fig 9).

Furthermore, based on the aforementioned mechanism, one may assume that in the presence of copper ions, formation of the thiol-copper-thiol bond between cysteine residues of several BSA molecules is feasible. In our previous work reported by Dashtestani, F. et al [40], we verified that cysteine could be co-ordinated to copper via its thiol functional group. Therefore, it can be proposed that ultrasonication has dual roles. Firstly, ultrasonication would be able to exclude the citrate layer around nanoparticles; the released citrate ions could then induce changing in the copper oxidation degree from zero to double positive ions. Secondly, the production of superoxide ions as a result of ultrasonication process, led to the formation of cysteinyl radicals. While, BSA is under ultrasonication in the presence of copper ions, cysteinyl radicals from adjacent BSA molecules bind each other via a copper bridge (thiol-copper-thiol) to form ACuNPs (Fig 9).

The mechanism of the toxicity of CuNPs involves rupturing of the cellular membrane [41]. Based on the fact that there is a higher O_2_ concentration in the cell membrane compared to the cell media, CuNPs are oxidized as they pass through the cell membrane, producing copper ions. Therefore, the rupturing of the cellular may be a result of the metal release process into the cell, leading to the production of H_2_O_2_ locally at the cell membrane [42]. It should be noted that rupturing of the cellular membrane is not only due to the released copper ion into the cell, but also due to the direct exposure of human alveolar epithelial cell line A549 to the copper ions, leading to less toxic damage to the membrane [4, 43]. It should be noted that copper ion release process from CuNPs into the vicinity of membrane surface is a critical stage in CuNPs toxicity. Other studies are based on carbon-coated CuNPs and CuCl_2_. Both of these components did not cause membrane rupture whereas CuNPs induced rupturing of the cell membrane [44]. It can be concluded that the ultimate fate of Cu-metal nanoparticles upon entering the body will be degrading and producing Cu^2+^ ions. Therefore, after having their cytotoxic effect on cancer cell and being released as ions, they will not be cytotoxic, because Cu^2+^ ion is not cytotoxic in concentrations lower than 500 μM.

Herein, we developed a novel nanocomposite of CuNPs covered by albumin as a nanocarrier and assayed its cytotoxic properties against invasive human cancer cell line of MDA-MB-231 for the first time. Our results showed that the delivery of copper nanoparticle with albumin nanocarrier, has an inhibitory effect on the viability of human breast cancer cell lines similar to the effect of naked copper nanoparticles. However, the IC_50_ value for ACuNPs was less than that of CuNPs. This phenomenon is probably due to enhancement of the albumin internalization in cancer cells [45] and subsequent increase of the intracellular levels of CuNPs. A principle reason for applying a cytotoxic chemotherapeutic agent is the potential of this agent to induce apoptosis and cell death in cancer cells [46]; since during apoptosis, the cells die without generating any inflammatory responses [47]. Statistics imply that breast cancer is faced with uncontrolled growth and there is an urgent need to discover and develop a new anticancer agent with fewer side effects for normal cells [48]. Thus, the potential of apoptosis induction by ACuNPs against invasive breast cancer cell lines was assayed. The cytomorphological changes under an inverted phase-contrast microscopy, acridine orange in combination with ethidium bromide staining and DNA fragmentation assay after 24 hours were used. According to the data analysis, ACuNPs had the potential to induce apoptosis in breast cancer cells. Treating cells with a variety of stress factors, such as anti-cancer drugs, lead to an increase in ROS levels and the cellular apoptosis [49] by triggering pro-apoptotic signaling molecules [50]. It is assumed that oxidative stress causing factor can selectively lead to cancer cells death compared with the normal cell, due to the production of higher concentration of ROS in cancer cells [48].

Finally, in vivo data clearly emphasized that ACuNPs has a positive effect on growth of BALB/c mice, because All groups receiving ACuNPs (included: groups 2, 4 and 5) had higher weight than non-receiving ones. Also, group 2 which was injected only by ACuNPs had the highest weight among all. The weight gain phenomenon after treatment with nanoparticle has been reported in some reports especially in which they use protein nanoparticle. Kishore Golla et al., also showed that when rats were treated by an anti-cancer drug (doxorubicin) loaded protein nanoparticle (apotransferrin and lactoferrin nanoparticles), a slight increase in body weight of was observed [51]. The probable reason for this phenomenon may be due to promoting diet and enriching it by protein. ACuNPs effects on both prevention and treatment of tumor glands, although it had better effect on treatment and removing glands.

## 5. Conclusion

A new nanocomposite of copper nanoparticle embedded in albumin nanocarrier was prepared (ACuNPs) which had the spherical shape with the smooth border and an average diameter under 100 nm. Structural studies revealed that during the process of ACuNPs synthesis, the structure of BSA remained almost intact. Biochemical studies showed that ACuNPs induces cell death in human breast cancer cells (MDA-MB 231); however, the degree of induction was dose-dependent. It was verified that apoptosis is the route of cell death in the treatment of MDA-MB-231 by ACuNPs based on the evident of (i) cytomorphological changes under an inverted phase-contrast microscopy, (ii) permeability of treated cell toward acridine orange and ethidium bromide, (iii) creating ladder pattern in DNA gel electrophoresis and (iv) quantitative data of flow cytometry. ACuNPs was also found to induce oxidative stress which was evident by ROS generation. Cytotoxicity of ACuNPs against breast cancer cells was 5.7 times more severe than that on normal cells. Over all, one may conclude that the ACuNPs nanocomposite can be a good candidate to be used as a chemotherapeutic agent against invasive breast cancer cells; however, further pharmacological studies are needed.

## Abbreviations

7-AAD: 7-amino-actinomycin
A549: adenocarcinoma's human alveolar basal epithelial cells
ACuNPs: albumin-CuNPs
AO/EB: acridine orange and ethidium bromide
BSA: bovine serum albumin
CD: circular dichroism
Cu: copper
CuNPs: copper nanoparticles
DCFH-DA: 2',7'-dichlorofluorescein diacetate
DLS: dynamic light scattering
DMEM: dulbecco’s modified eagle's medium
DMSO: dimethyl sulfoxide and trisodium citrate
gp60: an albumin-binding glycoprotein
HSA: human serum albumin
HuMEC: human mammary epithelial cells
IBRC: Iranian biological resource center
IC_50_: the half maximal inhibitory concentration
MCF-10A: non-tumourigenic human breast epithelial cells
MDA-MB 231 cells: human breast cancer cells
MTT: 3-(4,5-dimethyl thiazol)-2,5-diphenyl tetrazoliumbromide
ANPs: blank albumin nanoparticle
PBS: phosphate buffer saline
PI: propidium iodide
ROS: reactive oxygen species
TEM: transmission electron microscopy
THP-1: acute monocytic leukemia

## References

[1] Franco-Molina MA, Mendoza-Gamboa E, Sierra-Rivera CA, Gómez-Flores RA, Zapata-Benavides P, Castillo-Tello P, et al Antitumor activity of colloidal silver on MCF-7 human breast cancer cells. J Exp Clin Cancer Res. 2010;29(1):148. 21080962 10.1186/1756-9966-29-148 PMC2996348

[2] Liu H, Liu Y, Wang Z, He P. Facile synthesis of monodisperse, size-tunable SnS nanoparticles potentially for solar cell energy conversion. Nanotechnology. 2010;21(10):105707 doi: 10.1088/0957-4484/21/10/105707 20157232 10.1088/0957-4484/21/10/105707

[3] Sankar R, Maheswari R, Karthik S, Shivashangari KS, Ravikumar V. Anticancer activity of Ficus religiosa engineered copper oxide nanoparticles. Materials Science and Engineering: C. 2014;44:234–9. 25280701 10.1016/j.msec.2014.08.030

[4] Karlsson HL, Cronholm P, Gustafsson J, Möller L. Copper Oxide Nanoparticles Are Highly Toxic: A Comparison between Metal Oxide Nanoparticles and Carbon Nanotubes. Chemical Research in Toxicology. 2008;21(9):1726–32. doi: 10.1021/tx800064j 18710264 10.1021/tx800064j

[5] Lanone S, Rogerieux F, Geys J, Dupont A, Maillot-Marechal E, Boczkowski J, et al Comparative toxicity of 24 manufactured nanoparticles in human alveolar epithelial and macrophage cell lines. Particle and Fibre Toxicology. 2009;6(1):1–12. 19405955 10.1186/1743-8977-6-14 PMC2685765

[6] Midander K, Cronholm P, Karlsson HL, Elihn K, Möller L, Leygraf C, et al Surface Characteristics, Copper Release, and Toxicity of Nano‐and Micrometer‐Sized Copper and Copper (II) Oxide Particles: A Cross‐Disciplinary Study. Small. 2009;5(3):389–99. doi: 10.1002/smll.200801220 19148889 10.1002/smll.200801220

[7] Safavi M, Esmati N, Ardestani SK, Emami S, Ajdari S, Davoodi J, et al Halogenated flavanones as potential apoptosis-inducing agents: synthesis and biological activity evaluation. European journal of medicinal chemistry. 2012;58:573–80. doi: 10.1016/j.ejmech.2012.10.043 23174316 10.1016/j.ejmech.2012.10.043

[8] Lu Y, Mahato RI. Pharmaceutical perspectives of cancer therapeutics: Springer Science & Business Media; 2009.

[9] Meshkini A, Yazdanparast R. Involvement of oxidative stress in taxol-induced apoptosis in chronic myelogenous leukemia K562 cells. Experimental and Toxicologic Pathology. 2012;64(4):357–65. doi: 10.1016/j.etp.2010.09.010 21074392 10.1016/j.etp.2010.09.010

[10] Brodská B, Holoubek A. Generation of reactive oxygen species during apoptosis induced by DNA-damaging agents and/or histone deacetylase inhibitors. Oxidative medicine and cellular longevity. 2011;2011. 10.1155/2011/253529 PMC3178180 21949898

[11] Wartlick H, Spänkuch-Schmitt B, Strebhardt K, Kreuter J, Langer K. Tumour cell delivery of antisense oligonuclceotides by human serum albumin nanoparticles. Journal of Controlled Release. 2004;96(3):483–95. doi: 10.1016/j.jconrel.2004.01.029 15120904 10.1016/j.jconrel.2004.01.029

[12] Mirtsching B, Cosgriff T, Harker G, Keaton M, Chidiac T, Min M. A phase II study of weekly nanoparticle albumin-bound paclitaxel with or without trastuzumab in metastatic breast cancer. Clinical breast cancer. 2011;11(2):121–8. doi: 10.1016/j.clbc.2011.03.007 21569998 10.1016/j.clbc.2011.03.007

[13] Kim TH, Jiang HH, Youn YS, Park CW, Tak KK, Lee S, et al Preparation and characterization of water-soluble albumin-bound curcumin nanoparticles with improved antitumor activity. International journal of pharmaceutics. 2011;403(1):285–91. 21035530 10.1016/j.ijpharm.2010.10.041

[14] Wang Z-m, Ho JX, Ruble JR, Rose J, Rüker F, Ellenburg M, et al Structural studies of several clinically important oncology drugs in complex with human serum albumin. Biochimica et Biophysica Acta (BBA)-General Subjects. 2013;1830(12):5356–74. 23838380 10.1016/j.bbagen.2013.06.032

[15] Poór M, Li Y, Matisz G, Kiss L, Kunsági-Máté S, Kőszegi T. Quantitation of species differences in albumin–ligand interactions for bovine, human and rat serum albumins using fluorescence spectroscopy: A test case with some Sudlow's site I ligands. Journal of Luminescence. 2014;145:767–73.

[16] Yanase K, Arai R, Sato T. Intermolecular interactions and molecular dynamics in bovine serum albumin solutions studied by small angle X-ray scattering and dielectric relaxation spectroscopy. Journal of Molecular Liquids. 2014.

[17] Rashid MU, Bhuiyan MKH, Quayum ME. Synthesis of silver nano particles (Ag-NPs) and their uses for quantitative analysis of vitamin C tablets. Dhaka University Journal of Pharmaceutical Sciences. 2013;12(1):29–33.

[18] Desai NP, Tao C, Yang A, Louie L, Yao Z, Soon-Shiong P, et al Protein stabilized pharmacologically active agents, methods for the preparation thereof and methods for the use thereof. Google Patents; 2004.

[19] Zeinabad HA, Zarrabian A, Saboury AA, Alizadeh AM, Falahati M. Interaction of single and multi wall carbon nanotubes with the biological systems: tau protein and PC12 cells as targets. Scientific Reports. 2016;6:26508 doi: 10.1038/srep26508 27216374 10.1038/srep26508 PMC4877924

[20] Jafari Azad V, Kasravi S, Alizadeh Zeinabad H, Memar Bashi Aval M, Saboury AA, Rahimi A, et al Probing the conformational changes and peroxidase-like activity of cytochrome c upon interaction with iron nanoparticles. Journal of Biomolecular Structure and Dynamics. 2016:1–13. 10.1080/07391102.2016.1222972 27632558

[21] Fisher GA, Sikic BI. Clinical studies with modulators of multidrug resistance. Hematology/oncology clinics of North America. 1995;9(2):363–82. 7642468

[22] Kheirollahi A, Pordeli M, Safavi M, Mashkouri S, Naimi-Jamal MR, Ardestani SK. Cytotoxic and apoptotic effects of synthetic benzochromene derivatives on human cancer cell lines. Naunyn-Schmiedeberg's archives of pharmacology. 2014;387(12):1199–208. doi: 10.1007/s00210-014-1038-5 25261336 10.1007/s00210-014-1038-5

[23] LeBel CP, Ischiropoulos H, Bondy SC. Evaluation of the probe 2', 7'-dichlorofluorescin as an indicator of reactive oxygen species formation and oxidative stress. Chemical Research in Toxicology. 1992;5(2):227–31. 1322737 10.1021/tx00026a012

[24] Christian P, Bromfield M. Preparation of small silver, gold and copper nanoparticles which disperse in both polar and non-polar solvents. Journal of Materials Chemistry. 2010;20(6):1135–9.

[25] Díaz-Visurraga J, Daza C, Pozo C, Becerra A, von Plessing C, García A. Study on antibacterial alginate-stabilized copper nanoparticles by FT-IR and 2D-IR correlation spectroscopy. Int J Nanomedicine. 2012;7:3597–612. doi: 10.2147/IJN.S32648 22848180 10.2147/IJN.S32648 PMC3405878

[26] Hanaor D, Michelazzi M, Leonelli C, Sorrell CC. The effects of carboxylic acids on the aqueous dispersion and electrophoretic deposition of ZrO2. Journal of the European Ceramic Society. 2012;32(1):235–44.

[27] áO'Brien RW. Electroacoustic studies of moderately concentrated colloidal suspensions. Faraday Discussions of the Chemical Society. 1990;90:301–12.

[28] Sastry M, Mayya K, Bandyopadhyay K. pH Dependent changes in the optical properties of carboxylic acid derivatized silver colloidal particles. Colloids and Surfaces A: Physicochemical and Engineering Aspects. 1997;127(1):221–8.

[29] Ahmad A, Senapati S, Khan MI, Kumar R, Ramani R, Srinivas V, et al Intracellular synthesis of gold nanoparticles by a novel alkalotolerant actinomycete, Rhodococcus species. Nanotechnology. 2003;14(7):824.

[30] Zhao X, Liu R, Chi Z, Teng Y, Qin P. New insights into the behavior of bovine serum albumin adsorbed onto carbon nanotubes: comprehensive spectroscopic studies. The Journal of Physical Chemistry B. 2010;114(16):5625–31. doi: 10.1021/jp100903x 20373820 10.1021/jp100903x

[31] Hospes M, Hendriks J, Hellingwerf KJ. Tryptophan fluorescence as a reporter for structural changes in photoactive yellow protein elicited by photo-activation. Photochemical & Photobiological Sciences. 2013;12(3):479–88. 23183905 10.1039/c2pp25222h

[32] Pabbathi A, Patra S, Samanta A. Structural transformation of bovine serum albumin induced by dimethyl sulfoxide and probed by fluorescence correlation spectroscopy and additional methods. ChemPhysChem. 2013;14(11):2441–9. doi: 10.1002/cphc.201300313 23780704 10.1002/cphc.201300313

[33] Honary S, Zahir F. Effect of zeta potential on the properties of nano-drug delivery systems-a review (Part 2). Tropical Journal of Pharmaceutical Research. 2013;12(2):265–73.

[34] Ahmad J, Alhadlaq HA, Siddiqui MA, Saquib Q, Al-Khedhairy AA, Musarrat J, et al Concentration-dependent induction of reactive oxygen species, cell cycle arrest and apoptosis in human liver cells after nickel nanoparticles exposure. Environmental Toxicology. 2015;30(2):137–48. doi: 10.1002/tox.21879 23776134 10.1002/tox.21879

[35] Ahamed M, Akhtar MJ, Alhadlaq HA, Alshamsan A. Copper ferrite nanoparticle-induced cytotoxicity and oxidative stress in human breast cancer MCF-7 cells. Colloids and Surfaces B: Biointerfaces. 2016;142:46–54. doi: 10.1016/j.colsurfb.2016.02.043 26925725 10.1016/j.colsurfb.2016.02.043

[36] Nel A, Xia T, Mädler L, Li N. Toxic potential of materials at the nanolevel. Science. 2006;311(5761):622–7. doi: 10.1126/science.1114397 16456071 10.1126/science.1114397

[37] Hu R, Yong K-T, Roy I, Ding H, He S, Prasad PN. Metallic nanostructures as localized plasmon resonance enhanced scattering probes for multiplex dark-field targeted imaging of cancer cells. The Journal of Physical Chemistry C. 2009;113(7):2676–84. 10.1021/jp8076672 PMC2717617 20046993

[38] Fahmy B, Cormier SA. Copper oxide nanoparticles induce oxidative stress and cytotoxicity in airway epithelial cells. Toxicology In Vitro. 2009;23(7):1365–71. doi: 10.1016/j.tiv.2009.08.005 19699289 10.1016/j.tiv.2009.08.005 PMC2756312

[39] Desai NP, Tao C, Yang A, Louie L, Zheng T, Yao Z, et al Protein stabilized pharmacologically active agents, methods for the preparation thereof and methods for the use thereof. Google Patents; 1999.

[40] Dashtestani F, Ghourchian H, Eskandari K, Rafiee-Pour H-A. A superoxide dismutase mimic nanocomposite for amperometric sensing of superoxide anions. Microchimica Acta. 2015;182(5–6):1045–53.

[41] Karlsson HL, Cronholm P, Hedberg Y, Tornberg M, De Battice L, Svedhem S, et al Cell membrane damage and protein interaction induced by copper containing nanoparticles—Importance of the metal release process. Toxicology. 2013;313(1):59–69. doi: 10.1016/j.tox.2013.07.012 23891735 10.1016/j.tox.2013.07.012

[42] VanWinkle BA, De Mesy Bentley KL, Malecki JM, Gunter KK, Evans IM, Elder A, et al Nanoparticle (NP) uptake by type I alveolar epithelial cells and their oxidant stress response. Nanotoxicology. 2009;3(4):307–18. doi: 10.1080/17435390903121949 20563262 10.1080/17435390903121949 PMC2886975

[43] Midander K, Cronholm P, Karlsson HL, Elihn K, Möller L, Leygraf C, et al Surface Characteristics, Copper Release, and Toxicity of Nano- and Micrometer-Sized Copper and Copper(II) Oxide Particles: A Cross-Disciplinary Study. Small. 2009;5(3):389–99. doi: 10.1002/smll.200801220 19148889 10.1002/smll.200801220

[44] Minocha S, Mumper RJ. Effect of Carbon Coating on the Physico-chemical Properties and Toxicity of Copper and Nickel Nanoparticles. Small. 2012;8(21):3289–99. 10.1002/smll.201200478 22837153

[45] Trajkovska M. Macropinocytosis supports cancer cell proliferation. Nat Cell Biol. 2013;15(7):729-.

[46] Hannun YA. Apoptosis and the dilemma of cancer chemotherapy. Blood. 1997;89(6):1845–53. 9058703

[47] Satchell P, Gutmann J, Witherspoon D. Apoptosis: an introduction for the endodontist. International endodontic journal. 2003;36(4):237–45. 12702117 10.1046/j.1365-2591.2003.00657.x

[48] Parkin DM, Bray F, Ferlay J, Pisani P. Global cancer statistics, 2002. CA: a cancer journal for clinicians. 2005;55(2):74–108. 10.3322/canjclin.55.2.74 15761078

[49] Lampiasi N, Azzolina A, D'Alessandro N, Umezawa K, McCubrey JA, Montalto G, et al Antitumor effects of dehydroxymethylepoxyquinomicin, a novel nuclear factor-κB inhibitor, in human liver cancer cells are mediated through a reactive oxygen species-dependent mechanism. Molecular pharmacology. 2009;76(2):290–300. doi: 10.1124/mol.109.055418 19461054 10.1124/mol.109.055418

[50] Benhar M, Engelberg D, Levitzki A. ROS, stress‐activated kinases and stress signaling in cancer. EMBO reports. 2002;3(5):420–5. doi: 10.1093/embo-reports/kvf094 11991946 10.1093/embo-reports/kvf094 PMC1084107

[51] Golla K, Bhaskar C, Ahmed F, Kondapi AK. A target-specific oral formulation of doxorubicin-protein nanoparticles: efficacy and safety in hepatocellular cancer. Journal of Cancer. 2013;4(8):644 doi: 10.7150/jca.7093 24155776 10.7150/jca.7093 PMC3805992

